# The microRNA-127-3p directly targeting *Vamp2* in C2C12 myoblasts

**DOI:** 10.1080/19768354.2018.1512520

**Published:** 2018-08-25

**Authors:** Jie Li, Gaofu Wang, Jing Jiang, Lin Fu, Peng Zhou, Hangxing Ren

**Affiliations:** Herbivorous Livestock Institute, Chongqing Academy of Animal Sciences, Chongqing, People's Republic of China

**Keywords:** Myogenesis, miR-127-3p, *Vamp2*, C2C12

## Abstract

MicroRNAs (miRNAs) have been reported that can regulate skeletal muscle growth and development. Previously, we demonstrated that miR-127-3p were differently expressed in skeletal muscle and muscle cells. However, the molecular mechanism of miR-127-3p regulation of skeletal myogenesis are not well elucidated. In this study, we transfected miR-127-3p into C2C12 cells, and found miR-127-3p induces myogenesis by targeting *Vamp2*. Moreover, the regulatory mechanism of *Vamp2* in myoblasts proliferation and differentiation was further confirmed. In conclusion, our data providedevidences that miR-127-3p reciprocally regulated myoblasts proliferation and differentiation through directly targeting *Vamp2*.

## Introduction

MicroRNAs (miRNAs) are endogenous noncoding small RNAs of 18–25 nucleotides (nt) in length, regulating gene expression by translational inhibition or direct induction of mRNA degradation at posttranscriptional level (Chekulaevac and Filipowicz 2009). Many evidences indicated that miRNAs participated in almost all biological process including cell cycle, development, metabolism, diseases, and so on (Bushati and Cohen 2007). The skeletal myogenesis is a complicated multi-step process, which is not only orchestrated by a seriesof crucial coding-protein regulators such as *MyoD*, *MyoG*, *Myf5*, and *MRF4* (Singh and Dilworth 2013), but also is regulated by miRNAs, such as miR-1(Chen et al. 2006), miR-133 (Kim et al. 2006), miR-206; (Anderson et al. 2006) and so on.

MiR-127located within an miRNA cluster in the Dlk1-Dio3 region, it has been reported that involved with the development of cancer cells (Yang et al. 2013; Zhang et al. 2016), normal cell lineages such as embryonic stem cells (Ma et al. 2016), fibroblasts (Chen et al. 2013), and liver cells (Pan et al. 2012). These findings indicated the important roles of miR-127 in myogenesis cells and embryonic development. Most recently, Zhai et al. revealed miR-127 enhanced myogenic cell differentiation by targeting *S1PR3*, and overexpression of miR-127 in muscular dystrophy model mdx mice considerably ameliorated the disease phenotype (Zhai et al. 2017). Interestingly, the miR-127 precursor could transcribe two types of mature miRNAs (miR-127-3p and miR-127-5p), the specific function for either of miR-127 products remain to be elucidated in detail, respectively. In our study, we focused on the molecular mechanism of miR-127-3p in myogenesis. Our recent study indicated that miR-127-3p probably involves in the myoblasts proliferation and differentiation (Li et al. 2016). Nevertheless, the molecular mechanism of this microRNA acting on the myoblasts proliferation and differentiation is still unclear. In the present study, our general hypothesis was that miR-127-3p could regulate the proliferation and differentiation of C2C12 cells by targeting *Vamp2*. Our findings enrich the myogenic network involved in microRNAs, as well as provide some valuable information towards pathogenesis of muscle disease and gene-targeted therapy.

## Materials and methods

### Cell culture

C2C12 cells were maintained in Dulbecco’s modified Eagle’s medium (DMEM) (Gibco, Grand Island, USA) with 10% fetal bovine serum (FBS) at 37°C in a 5% CO_2_-humidified atmosphere. For myogenic differentiation, myoblasts were transferred to DMEM supplemented plus 2% horse serum (HS) when near-confluent cells rearched up to 80%. Replenished with fresh medium every 2 days.

293 T cells were cultured for the dual-luciferase activity assay. The cells were incubated in DMEM supplemented with 10% FBS, and the medium were changed every 2 days.

### Cell transfection

The overexpression (miR-127-3p mimic), knock-down (miR-127-3p inhibitor) and their own negative controls (NC) were purchased from Applied Biosystems (Foster City, CA, USA). The mimic (50nM), inhibitor (50nM) or negative controls (50nM) were transfected the cells under different conditions using Lipofectamine 2000 (Invitrogen, USA) according to the manufacturer’s instructions.

### Real time-PCR of mRNA and miRNA

Total RNA was extracted from cultured C2C12 cells using Trizol reagent (Invitrogen, USA), and the cDNA was synthesized by total RNA using Transcriptor First Strand cDNA Synthesis Kit (Roche, Germany). The expression levels of mRNA were detected using FastStart Universal SYBR Green Green Master (ROX) (Roche, Germany). For miRNA, total RNA was used to cDNA synthesis using TaqMan MicroRNA Reverse Transcription Kit (Applied Biosystems, USA). The quantitative real-time PCR (qRT-PCR) (Applied Biosystems, USA) was performed according to the manufacturer’s protocol. The miRNA expression was normalized against the expression of *U6*, whereas *GAPDH* was used as control gene to normalize the abundance of target mRNA expression. The mRNA primers information is shown in Table S.

### Dual-luciferase reporter assays

The WT 3′-UTR of *Vamp2*, containing the putatived miR-127-3p binding sites, and the Mut 3′-UTR of *Vamp2* with site-directed mutating the seed regions of the miR-127-3p binding sites were amplified from mouse DNA and inserted into the psiCHECK-2 vector (Promega, USA). 293 T or C2C12 cells seeded in 96-well plates at 1 × 10^3^ per well. Co-transfection of the dual-luciferase vector for *Vamp2* of WT and Mut into 293 T and C2C12 cells with the miR-127-3p mimic or negative control using Lipofectamine 2000 (Invitrogen, USA). Luciferase activity values were determined using the Dual-Luciferase Reporter Assay System (Promega, USA).

### Cell count and fusion index analysis

C2C12 cells proliferation was measured using Countess II FL automatic cell counter equipment to calculate cell count. In brief, cells were plated in 12-well plates at 1 × 10^4^ per well before transfection. After cultured for 72 h of proliferation, washed with PBS, 0.25%trypsin digested, terminated, and 10 µL mixture of cells were calculated by Countess II FL automatic cell counter equipment. Besides, fusion index was counted as the percentage of nuclei in myotubes with ≥ 2 nuclei/all of nuclei. Each data point was generated from randomly chosen microscopic fields containing in total 200 or more nuclei.

### Immunofluorescence analysis

C2C12 cells cultured in 12-well plates were used to immunofluorescence analysis. Principally, transfected with miRNA as described above, cells were washed with PBS, added work solution 1ug/ml DAPI (Roche, Germany) 15 mins, subjected to fluorescent microscopy. Fluorescence was detected with an Olympus microscope (FV1000, Olympus, Tokyo, Japan).The images were processed in Adobe Photoshop CS3.

### Statistical analysis

Each experiment was performed three times independently and each time with three replicates. All results were represented as mean ± S.E.M. Statistical analysis were performed by a Student’s t-test and statistical significance. *p *< 0.05 was considered threshold.

## Results

### MiR-127-3p directly targeted the 3′UTRs of *Vamp2*

**Figure 1.**
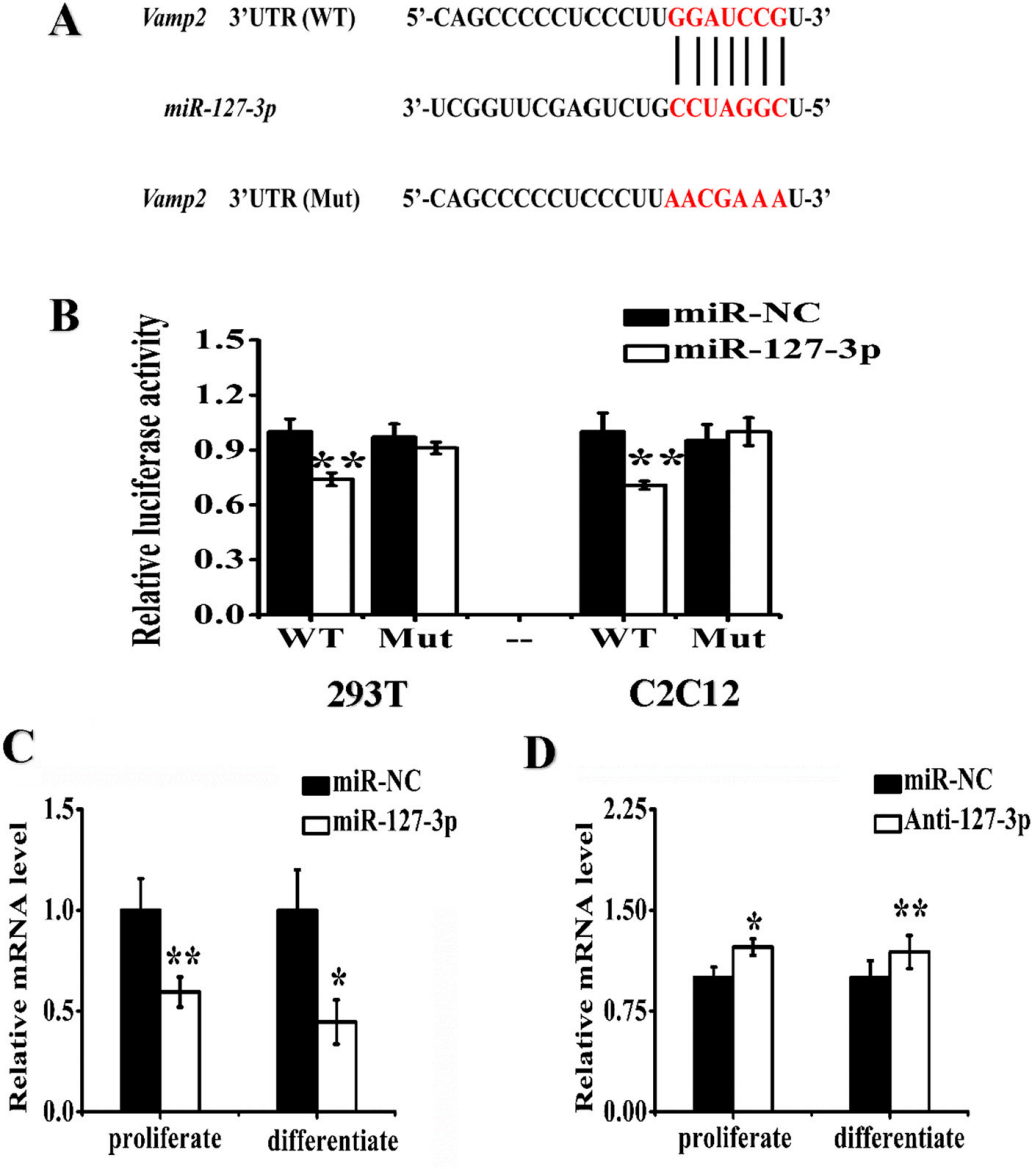
MiR-127-3p directly targets the 3’ UTR of *Vamp2*. (A) The illustration of target gene *Vamp2* wild-type (WT) or mutant (Mut) 3′-UTRs containing the putative miR-127-3p binding sites. (**B**) The 3’ UTR luciferase reporter vector of mouse *Vamp2* containing miR-127-3p targeting sites, were co-transfected with miR-127-3p mimic (or negative control) into 293 T cells and C2C12 cells, subjected to luciferase assays after thirty-six hours. The mRNA expression of *Vamp2* were detected by real-time qPCR in C2C12 cells after transfected with miR-127-3p mimic **(C)** and the inhibitor **(D)**. Quantitative data were represented as the mean ± S.E.M. *n* = 3. *P < 0.05, **P < 0.01.

At first, we predicted *Vamp2* may as the candidate target for miR-127-3p in the TargetScan database (http://www.targetscan.org) and StarBase 2.0 (Figure 1(A)). To validate the relationship between the miRNA and *Vamp2* , we co-transfected *Vamp2* with the miR-127-3p mimic or negative control into 293 T and C2C12 cells using the dual-luciferase vectors, respectively (Figure 1(B)). We found that miR-127-3p could effectively inhibited expression of wild-type *Vamp2* by combinating with its 3′-UTR, in contrast to the mutant *Vamp2*. These findings illustrated *Vamp2* might be the direct target for miR-127-3p.

Based on the miR-127-3p targeting *Vamp2*, we measured the mRNA level of *Vamp2* after transfection of miR-127-3p mimic or inhibitor into both C2C12 lines. Our results demonstrated that over-expression of miR-127-3p led to considerable decrease of *Vamp2* mRNA in proliferating and differentiating C2C12 cells (Figure 1(C)). When miR-127-3p was knocked-down by specific inhibitor, the mRNA levels of *Vamp2* were significantly increased in both C2C12 cells line (Figure 1(D)). These results indicated that the miR-127-3p negatively regulated *Vamp2* during myogenesis.

### The regulatory mechanism of *Vamp2* in myoblasts proliferation and differentiation

**Figure 2.**
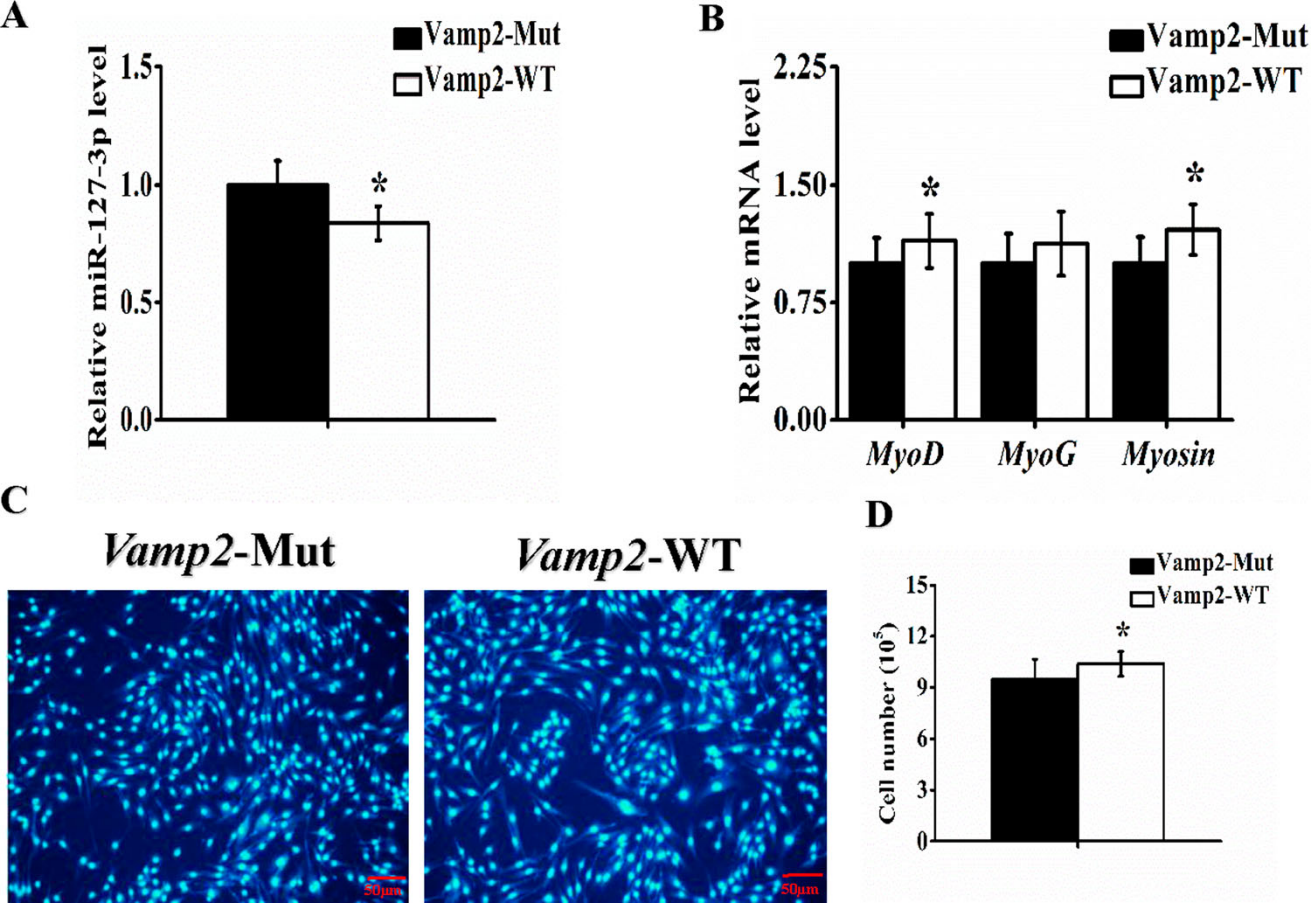
*Vamp2* participated in myoblasts proliferation. C2C12 cells were transfected with vector of *Vamp2* wild-type (WT) or mutant (Mut), then cultured for 72 h of proliferation. The relative expression of miR-127-3p **(A)** and myogenic marker genes *MyoD*, *MyoG* and *Myosin***(B)** were detected by real-time qPCR. **(C)** The nuclei of C2C12 cells were positive for DAPI (blue). Scale bar = 100 µm. **(D)** The cell count was calculated after transfected with *Vamp2* wild-type (WT) or mutant (Mut). statistical data were represented as the mean ± S.E.M. *n* = 6. Quantitative data were represented as the mean ± S.E.M. *n* = 3. *P < 0.05, **P < 0.01.

To further validate the regulatory action of miR-127-3p on *Vamp2*, we transferred the proliferating and differentiating C2C12 myoblasts with the wild-type (WT) or mutant (Mut) *Vamp2* in 3′-UTR in miR-127-3p binding sites, respectively. Cells were culturedin growth medium (GM) and transfected with the wild-type (WT) or mutant (Mut) *Vamp2*. Subsequently, the cell count and expression in myogenic marker genes were performed in the wild-type and the mutant. After 72 h incubation, the myogenic marker genes including *MyoD*, *MyoG*, and *Myosin* were high expressed in the wild-type *Vamp2* compared to the mutant by real-time qPCR (Figure 2(B)). Similarly, the cell count was significantly raised in the wild-type compared to the mutant (Figure 2(C-D)). These findings suggested *Vamp2* participated in C2C12 cells proliferation.

**Figure 3.**
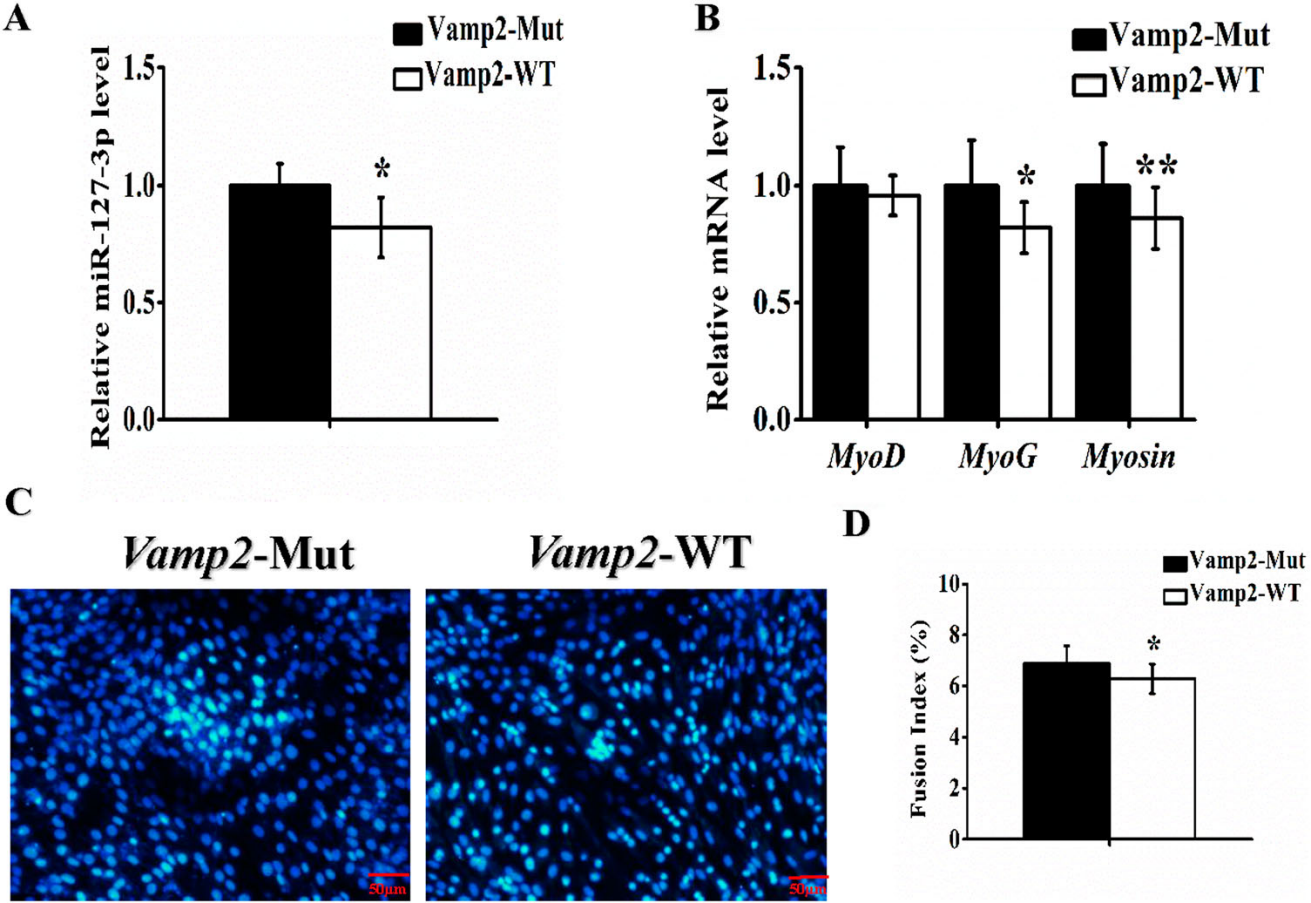
*Vamp2* involved in C2C12 myogenic differentiation. C2C12 cells were transfected with *Vamp2* wild-type (`WT) or mutant (Mut), induced to myogenic differentiation. At the 72 h of differentiation: The relative expression of miR-127-3p **(A)** and myogenic marker genes *MyoD*, *MyoG* and *Myosin***(B)** were detected by real-time qPCR. **(C)** The morphological features of myotubes in C2C12 cells were stained with DAPI (blue). Scale bar = 200 µm. The fusion index **(D)** were quantified in differentiating C2C12 cells. Statistical data were represented as the mean ± S.E.M. *n* = 6. Quantitative data were represented as the mean ± S.E.M. *n* = 3. *P < 0.05, **P < 0.01.

Besides, we transfected the wild-type (WT) or mutant (Mut) *Vamp2* into C2C12 myoblasts. Cells were induced to differentiate in differentiation medium (DM) with 80% confluence. Fusion index and gene expression were examined in the wild-type (WT) or mutant (Mut) *Vamp2*, respectively. We detected the significantly low mRNA levels of myogenic marker genes in the wild-type (WT) (Figure 3(B)), which was consistent with the lower fusion index in the wild-type (WT) team, compared with the findings of the mutant (Mut) (Figure 3(C-D)). The study confirmed the *Vamp2* involved in myoblasts myogenic differentiation.

## Discussion

Some studies have revealed the functional significance of miRNAs in regulating myogenic cells activation, proliferation, differentiation and fusion (Horak et al. 2016). Recently, Zhai et al. first identified the *S1PR3* gene as a direct target of miR-127, and it is mechanistically involved in miR-127-mediated satellite cells (SC) differentiation and muscle regeneration (Zhai et al. 2017). But the miR-127 precursor can transcribe two different types of mature miRNAs (miR-127-3p and miR-127-5p). The role of miR-127 on muscular dystrophy in mdx mice may be the mixed effects of miR-127-3p and miR-127-5p. In this report, we demonstrated that miR-127-3p regulated myoblasts proliferation and differentiation by targeting *Vamp2*, enrich the myogenic network of miRNAs. It also suggested that miR-127-3p as a potential therapeutic target in the treatment of human muscular diseases.

Vesicle-associated membrane protein 2 (*Vamp2*)/synaptobrevin, the prototypical v-SNARE, was first identified in synaptic vesicles from rat brain (Ralston et al. 1994), and is implicated in synaptic vesicle docking and fusion with plasma membrane proteins (Tajika et al. 2007). *Vamp2* was expressed in non-neuronal tissue (Watson et al. 2004), myofibers (Cain et al. 1992), adipocytes (Baumert et al. 1989), and quiescent satellite cells (Rossetto et al. 1996). In adult myocytes and adipocytes, *Vamp2* could regulate the GLUT4 translocation (Tajika et al. 2007). However, few studies on myogenic role of *Vamp2* have been reported up to now. Here, we revealed the myogenic role of *Vamp2* was controlled by miR-127-3p, and identified regulatory mechanism of *Vamp2* in myoblasts proliferation and differentiation. Intriguingly, myogenic miR-208b/499 were encoded by host genes intron of α-MHC, β-MHC and Myh7b, mature miRNAs also conversely regulate the expression content of myosin (Mccarthy et al. 2009). Relationship of feedback regulation between intron transcripts and their host genes further implies the complexity of expression regulation in mammal genome. In the present study, overexpression of miR-127-3p decreased mRNA level of *Vamp2* in C2C12 cells (Figure 1(C)), whereas overexpression of *Vamp2* also reduced level of miR-127-3p in myoblasts (Figures 2 and 3(A)). It seems that there is a feedback regulation between miR-127-3p and its target gene *Vamp2*. Interestingly, our subsequently bioinformatical analysis demonstrated that miR-127-3p was not encoded by *Vamp2* gene (data not shown), thusthe mechanism of feedback regulation of miR-127-3p by *Vamp2* may different from that of miR-208b/499, and it remains to be investigated in future. In conclusion, our findings not only contribute to molecular mechanisms of miR-127-3p in regulation of muscle development and growth, but also improved understanding of pathogenesis of human muscle disease and gene-targeted therapy.

## Supplementary Material

Supplementary_Materials1.pdf
