# Supplementary material for: The microRNA-127-3p directly targeting Vamp2 in C2C12 myoblasts
Source: Anim Cells Syst (Seoul). 2018 Aug 25;22(5):299–304. doi: 10.1080/19768354.2018.1512520 (PMC6171451; doi:10.1080/19768354.2018.1512520)
Supplement: Supplementary_Materials1.pdf [file TACS_A_1512520_SM1926.pdf]

Gene	Primer sequence (5’-3’)	Size (bp)
MyoD	F:GGCTCTCTCTGCTCCTTTGA<br>R:GTAGGGAAGTGTGCGTGCTC	149
MyoG	F:GCAGGCTCAAGAAAGTGAATG<br>R:AGGCGCTCAATGTACTGGAT	121
Myosin	F: AGGCACCTGCTGAAGAAGAC<br>R: CCTCGAAGGTCTGTGACTCC	100
Vamp2	F:GCTGGATGACCGTGCAGAT<br>R:GATGGCGCAGATCACTCCC	130
GAPDH	F:TCAAGAAGGTGGTGAAGCAG<br>R:AGGTGGAAGAGTGGGAGTTG	111
